# Infections Following Orthotopic Liver Transplantation

**DOI:** 10.1155/1991/97375

**Published:** 1991

**Authors:** Paul M. Arnow

**Affiliations:** Department of Medicine Section of Infectious Diseases University of Chicago 5841 S. Maryland Avenue Chicago Illinois 60637 USA

## Abstract

The epidemiology of infections associated with orthotopic liver transplantation is summarized herein, and approaches to prophylaxis are outlined. Infection is a major complication following orthotopic liver transplantation, and more than half of transplant recipients develop at least one infection. The risk of infection is highest in the first month after transplantation, and the most common pathogens are bacteria and cytomegalovirus (CMV). Bacterial infections usually occur in the first month, arise in the abdomen, and are caused by aerobes. The peak incidence of CMV infection is late in the first month and early in the second month after transplantationn. CMV syndromes include fever and neutropenia, hepatitis, pneumonitis, gut ulceration, and disseminated infection. Other significant problems are *Candida* intraabdominal infection, *Herpes simplex* mucocutaneous infection or hepatitis, adenovirus hepatitis, and *Pneumocystis carinii* pneumonia.

Prophylaxis of infection in liver transplant recipients has not been well-studied. Several different regimens of parenteral, oral absorbable, and/or oral non-absorbable antibiotics active against bacteria and yeast have been used at various centers, but no randomized controlled trials have been conducted. Selective bowel decontamination appears to be a promising approach to the prevention of bacterial and *Candida infections*, while oral acyclovir may be a relatively convenient and effective agent for CMV prophylaxis.


*HPB Surgery*, 1991, Vol. 3, pp. 221-233
Reprints available directly from the publisher
Photocopying permitted by license only

© 1991 Harwood Academic Publishers GmbH
Printed in the United Kingdom

### REVIEW ARTICLE

### INFECTIONS FOLLOWING ORTHOTOPIC LIVER TRANSPLANTATION

PAUL M. ARNOW
*Department of Medicine, Section of Infectious Diseases, University of Chicago, 5841 S. Maryland Avenue, Chicago, Illinois 60637*

*(Received 3 August 1990)*

The epidemiology of infections associated with orthotopic liver transplantation is summarized herein, and approaches to prophylaxis are outlined. Infection is a major complication following orthotopic liver transplantation, and more than half of transplant recipients develop at least one infection. The risk of infection is highest in the first month after transplantation, and the most common pathogens are bacteria and cytomegalovirus (CMV). Bacterial infections usually occur in the first month, arise in the abdomen, and are caused by aerobes. The peak incidence of CMV infection is late in the first month and early in the second month after transplantationn. CMV syndromes include fever and neutropenia, hepatitis, pneumonitis, gut ulceration, and disseminated infection. Other significant problems are *Candida* intraabdominal infection, *Herpes simplex* mucocutaneous infection or hepatitis, adenovirus hepatitis, and *Pneumocystis carinii* pneumonia.

Prophylaxis of infection in liver transplant recipients has not been well-studied. Several different regimens of parenteral, oral absorbable, and/or oral non-absorbable antibiotics active against bacteria and yeast have been used at various centers, but no randomized controlled trials have been conducted. Selective bowel decontamination appears to be a promising approach to the prevention of bacterial and *Candida* infections, while oral acyclovir may be a relatively convenient and effective agent for CMV prophylaxis.

Infection is a frequent complication of orthotopic liver transplantation (OLT), and most recipients experience at least one infection during the first few months after transplantation[1–4]. The high rate of infection is attributable in part to the poor underlying condition of patients who require transplantation, but the most important factors appear to be intra- and post-operative events. Transplantation surgery is characterized by a duration often exceeding seven hours[1,5–7], blood component replacement exceeding two blood volumes[1,2,5–7], periods of hypotension, enterotomy (accidental or for choledochojejunostomy), and post-operative bleeding with hematoma formation. Surgery is followed by immunosuppression with several agents — e.g., cyclosporine, prednisone, and azathiaprine — to which bursts of high dose corticosteroids or anti-lymphocyte antibodies are added when rejection worsens. Under these conditions, it is not surprising that infection is a major problem.

Epidemiologic studies have characterized the frequency of infection, identified the major pathogens, and assessed possible risk factors for infection[1–5,8]. The results of these studies, summarized in this review, provide a useful guide to the clinician considering either empiric treatment of suspected infection or approaches to prophylaxis.

### BACTERIAL INFECTION

### *Infection Rates*

Rates of bacterial infection following OLT vary considerably, as shown by the results from selected centers summarized in Table 1. The average number of infections ranged from 0.51 to 2.0 infection per patient, and the percentage of patients experiencing at least one bacterial infection ranged from 36% to 68%. Differences in rates probably are attributable to several factors. First, definitions of infections sometimes were not stated and apparently varied. One group apparently included positive cultures of vascular catheters, bile, and urine as infections[3], while another group included only major infections having serious associated morbidity in most of their tabulations[4]. Second, patient populations may have differed in terms of underlying hepatic disease and in severity of illness at the time of transplantation. These characteristics were not described in some series, even though a possible role is suggested by the finding at one center that infection rates were higher in patients with hepatocellular disease than in those with cholestatic disease[1]. Third, bacterial prophylaxis differed at the various centers. Four centers administered intravenous cefotaxime, but the regimens varied in cefotaxime duration, choice of other systemic antibiotics, and use of oral agents for bowel decontamination. The center with the second highest rate of bacterial infection used only perioperative cefoxitin. Adoption of a selective bowel decontamination (SBD) regimen by the center reporting the lowest rate of bacterial infection may be a significant advance[9]. SBD regimens utilize oral non-absorbable agents selected for their activity against aerobic enteric gram-negative bacilli and *Candida*. These regimens have limited activity against gut anaerobes, whose preservation can impede colonization of the alimentary tract by the more pathogenic aerobes that have been important pathogens in infections following OLT[10].

### *Epidemiology*

Most bacterial infections occurred in the first month after OLT[1-5], and the incidence of bacterial infection declined steadily thereafter. The most common site of infection was the abdomen, as shown in Table 2. Other major sites of infection were primary bacteremia, pneumonia, and surgical wound infection. The surgical procedure itself and technical complications account for the predominant role of abdominal infections. In one series, episodes of peritonitis were associated with biliary leaks, cholangitis was associated with biliary duct stricture, and liver abscesses were associated with technical problems involving the implanted liver[1].

Bloodstream infections for which no primary source was apparent ranked second in frequency. It has been speculated that these infections originate in the abdomen[1,5], presumably as a consequence of unrecognized primary infection or bacterial translocation. Secondary bacteremia, typically arising from an identified abdominal site[1,4,5], also occurred commonly but is not listed in Table 2. Bacterial pneumonia was a less frequent problem, but in one series the mortality was 40%[1].

**Table 1** Rates of Bacterial Infection in Liver Transplant Recipients

| Centre | Authors | No. of patients | Rates of Bacterial Infection: % of patients with infection | Infections per patient | Infections per transplant | Antibiotic Prophylaxis |
|---|---|---|---|---|---|---|
| Pittsburgh | Kusne et al (1) | 101 | 59 | 0.89 | 0.71 | peri-operative ampicillin + cefotaxime × 5 d |
| UCLA | Colonna et al (2) | 35 | NR | 1.06 | 0.88 | pre-operative erythromycin + neomycin, and peri-operative ampicillin + cefotaxime × 5 d |
| Minnesota* | Ascher et al (3) | 93 | NR | 2.0 | 1.9 | peri-operative cefotaxime until extubation, then post-operative trimethoprim-sulfamethoxazole |
| Mayo Clinic† | Paya et al (4) | 53 | 36 | 0.51 | 0.51 | polymyxin E + gentamicin + nystatin beginning pre-operatively and continuing through post-operative day 21, and peri-operative cefotaxime + tobramycin × 2 d |
| Chicago | George et al (5) | 79 | 68 | 1.46 | 1.12 | peri-operative cefoxitin |

NR = not reported

* The criteria used to define infection were not stated, and infection apparently was diagnosed at some sites based only on positive cultures

† Infection rates are shown only for patients who required a single transplant, and minor infections are not included

**Table 2** Distribution of Bacterial Infections by Body Site

| Centre | Authors | *Percent of Serious Bacterial Infections at Specified Site* Abdomen* | Bloodstream† | Lungs | Wound | Other‡ |
|---|---|---|---|---|---|---|
| Pittsburgh | Kusne et al (1) | 41% | 9% | 19% | 10% | 21% |
| UCLA | Colonna et al (2) | 27% | 16% | 0 | 22% | 45% |
| Minnesota | Ascher et al (3) | 27% | 23% | 7% | 2% | 41% |
| Mayo Clinic** | Paya et al (4) | 48% | 19% | 15% | 0 | 18% |
| Chicago | George et al (5) | 30% | 23% | 10% | 17% | 20% |

* Infections of the abdomen include intraabdominal abscess, liver abscess, peritonitis, and cholangitis
† Bloodstream infections shown for Pittsburgh, UCLA, Mayo Clinic, and Chicago represent cases of bacteremia for which no primary site was identified; cases of secondary bacteremia and IV catheter sepsis are excluded
‡ Infections in this category include cases of IV catheter sepsis, urinary tract infection, colitis, pharyngitis, and meningitis
** The distribution shown is for major infections only

Pneumonia or cholangitis accounted for most bacterial infections occurring late after transplantation[1,4].

The predominant bacterial pathogens were enteric aerobic gram-negative bacilli and gram-positive cocci[1–5]. The relative frequency of isolation of these groups of organisms reflected substantially the antimicrobial prophylaxis regimen. Aerobic enteric gram-negative bacilli predominated except in patients at Mayo Clinic, where an SBD regimen was used[9]. Notably, *Pseudomonas aeruginosa* was an important pathogen both with conventional prophylaxis regimens[1–3,5] and SBD[4]. Anaerobes have been relatively infrequent pathogens, even though enterotomy is performed and the regimens used for prophylaxis generally did not include agents with potent activity against anaerobes. The highest proportion of infections from which anaerobes were isolated was 22% (6 of 27), reported in the series of patients receiving selective bowel decontamination[4].

### *Risk Factors*

Three centers have examined risk factors for infection after OLT. Colonna *et al.*[2] studied 35 patients who received 42 liver transplants at UCLA. Patients who developed infection due to any pathogen (bacterial, viral, or fungal) were compared by univariate analysis to uninfected patients in terms of 14 pre-, intra-, and post-operative variables. Infections occurring late after transplantation were included in the analysis. Risk of infection, due to any type of pathogen, was significantly associated with the following six variables: (1) age > 20 years, (2) biliary atresia, (3) lower pre-operative albumin level, (4) gastrointestinal or vascular complications, (5) requirement for post-operative hemodialysis, and (6) longer ICU stay after surgery. The authors did not examine which, if any, of these variables were risk factors for bacterial infection alone. Also, inclusion of late infections may have confounded the analysis of perioperative variables, because of the long interval between presumed cause (peri-operative variable) and the later effect (infection). Finally, the temporal sequence between each of the last two variables (dialysis and ICU stay) and infection is not noted, making the direction of the association uncertain.

Cuervas-Mons *et al.*[8] and Kusne *et al.*[1] examined sequential groups of patients at University of Pittsburgh. The first set of authors studied 93 patients who underwent OLT during the 3 year period ending in January 1984. Twenty-seven pre-operative variables were evaluated for their association with major bacterial infection within 60 days after OLT. The following five variables were significantly associated with infection: serum creatinine ≥ 1.52 mg/dl, neutrophil count ≥ 4,847 cells/mm$^3$, serum IgG ≥ 1.546 mg/dl, serum bilirubin ≥ 18.28 mg/dl, and WBC count ≥ 7,211 cells/mm$^3$. A mechanism for a causal association of these variables with infection was not proposed. Kusne *et al.*[1] subsequently reviewed 101 patients who underwent OLT at University of Pittsburgh between July 1984 and September 1985. Numerous pre-transplant, surgical, and post-operative variables were evaluated for an association with severe infection occurring early or late after transplantation. Variables associated with severe infection due to any pathogen (bacterial, viral, fungal, or protozoan) were then examined for an association with bacterial infection alone. The authors found that increased operative time (> 12 hours), increased antibiotic therapy (> 5 days) during the first three months after OLT, and increased transfusion of red blood cells (> 25 units) or fresh frozen plasma (> 30 units) were associated with bacterial infection. No association was found with the five pre-operative variables implicated by Cuervas-Mons *et al*[8].

George *et al.*[5] examined 19 possible risk factors for bacterial infection during the first two weeks after transplantation. Logistic regression analysis showed increased duration of transplantation surgery (≥ 8 hrs) and markedly elevated total serum bilirubin (≥ 12 mg/dl) to be significant risk factors.

In summary, the three studies of risk factors for bacterial infection after transplantation differed in design and results[1,5,8]. Although unconfirmed, the most plausible findings are that increased operative time and increased requirement for blood component transfusion may be risk factors for bacterial infection following OLT. These characteristics would seem to identify technically more difficult surgical procedures from which more complications might ensue.

### VIRAL INFECTION

### *Cytomegalovirus*

In liver transplant recipients, cytomegalovirus (CMV) is the most frequent cause of serious viral infection and often is the most common individual pathogen of any type[2–4,11]. Clinically significant CMV infection has been reported in about one-fourth of patients[1,2,4,11–14], as summarized in Table 3. An additional one-fourth of patients have serological evidence of CMV infection that is asymptomatic[4,11,12]. CMV infection has been detected as early as the first week after transplantation[4], but most cases occurred late in the first month or early in the second month following transplantation[4,11–14].

The clinical manifestations of CMV infection are diverse, and each manifestation has a spectrum of severity. The most common presentation is a "viral syndrome" characterized by fever and at least one of the following: atypical lymphocytes, neutropenia, or thrombocytopenia[1,11]. This syndrome generally resolves spontaneously or with reduced immunosuppression, but progression to organ involvement may occur[4,12]. In liver transplant recipients, the major CMV-infected organs are liver, gut, and lung. Perhaps because biopsies of transplanted livers routinely are obtained, hepatitis is the most commonly recognized CMV target organ syndrome[4,14,15]. The severity of CMV hepatitis is variable and in most cases mild[15].

**Table 3** Frequency of Cytomegalovirus (CMV) Infection in Liver Transplant Recipients

| *Centre* | *Authors* | *No. of Patients* | *No. (%) of Patients with Symptomatic CMV Infection* | *No. (%) of Patients with Asymptomatic CMV Infection* |
|---|---|---|---|---|
| Mayo Clinic | Paya et al (4) | 53 | 18 (34) | 12 (23) |
| UCLA | Colonna et al (2) | 35 | 6 (17) | NR* |
| Pittsburgh | Kusne et al (1) | 101 | 22 (22) | NR* |
| Pittsburgh | Singh et al (11) | 93 | 27 (29) | 28 (30) |
| Pittsburgh† | Breinig et al (12) | 43 | 5 (12) | 8 (19) |
| Cleveland | Gorensek et al (13) | 33 | 9 (27) | 7 (21) |
| Omaha | Stratta et al (14) | 211 | 73 (35) | NR* |

* NR = not reported
† This series included only pediatric patients

However, hepatitis also has been noted to be a feature of disseminated CMV infection[1,16]. Similarly, gastrointestinal involvement by CMV is common when cultures routinely are obtained[17]. Despite occasional cases of gastritis or gut ulceration and hemorrhage[11,12,18], the clinical consequences of gastrointestinal CMV usually are minimal[17]. CMV pneumonitis, alone or in the setting of disseminated infection, typically is diagnosed when significant interstitial infiltrates are present to warrant bronchoscopy or biopsy. The severity of illness at the time of diagnosis may account at least in part for the greater number of deaths due to pulmonary or disseminated CMV infection[1,18]. CMV infection also has been associated, most significantly as a cofactor with HLA antigens, in the development of vanishing bile duct syndrome[19].

The risk of CMV infection has been linked to the serostatus of the recipient and donor. When the recipient is seropositive, the rate of CMV infection (symptomatic or asymptomatic) typically exceeds 50%[11,12,14,15,20] and may be higher when the donor also is seropositive[14,15,20]. Evidence from kidney transplant recipients indicates that seropositive recipients can become infected with donor virus[20]. When the recipient is seronegative, the risk of infection has ranged from about 8% with a seronegative donor to nearly 100% with a seropositive donor[14,15,20]. The use of antilymphocyte therapy has been found to significantly increase the risk of subsequent CMV infection in one study[14]. However, in another study neither level of immunosuppressive therapy nor requirement for rejection therapy (which sometimes included antilymphocyte preparations) was significantly associated with CMV infection[13].

Administration of ganciclovir and temporary reduction or discontinuation of immunosuppressive therapy currently are the mainstays of treatment of CMV infection in liver transplant recipients. A favorable response has been reported in most patients considered to have severe infection[14,22,23], so additional therapies such as CMV immune globulin have not been routinely employed. Prophylaxis of CMV infection has been attempted primarily in bone marrow transplant recipients whose morbidity and mortality from CMV infection is greater than in other immunosuppressed patients. A reduced risk of infection and disease has been reported using CMV immune globulin and seronegative blood products[24] and using intravenous acyclovir[25]. In renal transplant recipients, high dose oral acyclovir also has been reported to reduce the rate of CMV infection and disease[26]. Advantages of oral acyclovir include reduced toxicity, lower cost, and greater ease of administration. Intravenous immune globulin plus intravenous then oral acyclovir is being used in at least one center for CMV prophylaxis[14]; use of ganciclovir for prophylaxis has not yet been reported.

*Other Viruses*

Other herpes viruses cause infections that are less frequent or less serious problems than CMV. Herpes simplex virus (HSV) reactivation results in mucocutaneous lesions (oral or genital) in about one-third of adult liver transplant recipients[1,11,20]. Because the prevalence of latent HSV infection is lower in children, their rate of post-transplant HSV infection is correspondingly lower[12,27]. Lesions typically have appeared about three weeks after transplantation[11,12,27], and treatment with acyclovir has been highly effective[1,12,27]. HSV hepatitis is an infrequent though life-threatening infection, sometimes occurring in association with extensive visceral involvement[1]. In one study in which pre-transplant serostatus was documented, the single case of HSV hepatitis represented primary HSV infection apparently acquired from the donor[11]. Varicella-zoster virus (VZV) infection, typically appearing in reactivation form as localized (dermatomal) zoster, has been reported months-to-years after transplantation in 5–13% of patients[1,11,20]. This infection generally is not serious, in contrast to varicella, or primary VZV infection, which may be fatal[11,12]. Epstein-Barr virus (EBV) infection, occurring either as primary or reactivation infection, is common and usually asymptomatic[11,12]. Hepatitis has been ascribed to EBV infection[27,28], and cases of EBV-induced lymphoproliferative syndrome have been reported as a late sequela[11,28,29].

Adenoviral infections have been a complication of OLT, primarily in children[27,30]. In the largest series reported, 22 (8%) of 262 children developed adenoviral infection, and five had hepatitis caused by adenovirus serotype 5[30]. Although the source of virus was not explored in these cases, the donor liver was implicated in a case from another center[2]. Interestingly, that case also was caused by adenovirus serotype 5.

Viral hepatitis recurring after transplantation has been a problem most notably with hepatitis B but with other agents as well. Graft reinfection with hepatitis B almost invariably occurs and has been associated sometimes with clinically evident hepatitis[31–33] and with impaired survival[34]. Passive immunoprophylaxis with hepatitis B immune globulin and immune modulation with interferon alfa have not been shown to be of benefit in preventing reinfection[32,33,35,36]. Nonetheless, liver transplantation remains a viable option for patients with liver failure due to chronic hepatitis B infection because of the favorable outcome in most patients followed for several years after transplantation[32,34]. Delta virus coinfection with hepatitis B also recurs after transplantation[32,37]; the number of patients reported to-date is too limited to conclude whether the outcome is significantly worse than with hepatitis B infection alone. Hepatitis A[38] and non-A, non-B hepatitis[39,40] also appear to recur after transplantation. A serious complication associated with acute or recurrent non-A, non-B hepatitis following transplantation is aplastic anemia which sometimes has been of prolonged duration[41].

The consequences of human immunodeficiency virus (HIV) infection in liver transplant recipients have been of concern, and the outcome in 15 patients has been reported by Tzakis *et al.*[42]. Seven patients were infected prior to transplantation, and eight seroconverted after transplantation. During a mean follow-up period of 4.5 years, four of the HIV infected patients developed manifestations of AIDS. However, overall survival of HIV infected patients was only slightly lower than that of other liver transplant recipients.

### FUNGAL INFECTION

Fungal infection in liver transplant recipients usually has been caused by *Candida*. The incidence of *Candida* infection has ranged from 13–35% at centers where no anti-fungal prophylaxis was given or where prophylaxis was begun at the time of transplantation[1,2,18,42–44]. In striking contrast, Paya *et al.*[4] reported only a 2% incidence of invasive *Candida* infection in a series of 53 patients given a selective bowel decontamination regimen which included nystatin. The regimen was given for a prolonged period beginning three days prior to active donor search and continuing until 21 days after transplantation[9]. The majority of *Candida* infections have occurred in the first month after transplantation, and virtually all cases have occurred within the first two months[1,4,18,42,43]. The usual site of infection has been the abdomen, and other presentations have included pneumonia, candidemia, and disseminated candidiasis[1,2,4,18,42,43]. Risk factors for fungal infection were examined among patients in two different time periods by investigators from University of Pittsburgh[1,42,43]. In both series, patients with candidiasis predominated, but they were pooled together with patients with aspergillosis. Risk factors identified in both studies were longer duration of systemic antibiotic therapy after transplantation, longer operative time, and greater requirement for post-transplantation surgery. In another study, recovery of *Candida* from surveillance cultures of multiple mucosal surfaces was a common finding in patients with invasive candidiasis but was of fairly low (47%) positive predictive value[44].

Other fungi of importance are *Aspergillus* and *Cryptococcus*. A few cases of infection due to these pathogens have been noted in most large series[1,3,4,27,42,43], but time of onset and other details of individual cases seldom are reported. Surprisingly, in one early series, aspergillosis was proven, usually at autopsy, in 9 (9%) of 100 patients[45]. Aspergillosis has presented most commonly as pneumonia or disseminated infection; involvement of brain has been a frequent finding in patients with disseminated infection[46] and also has been noted as a localized finding or with pulmonary infection[45–47]. Mortality is very high, so aspergillosis has accounted for a substantial proportion (about 20%) of cases in autopsy-based series of liver transplant recipients[46,47]. Conventional treatment of aspergillosis is with high dose amphotericin B, to which flucytosine or rifampin may be added. Itraconazole is a promising new agent that has been used successfully to treat solid organ transplant recipients with aspergillosis[48]. Cryptococcosis, an infrequent problem, has presented as meningitis or pneumonia. As in other immunocompromised patients, crypotoccal meningitis may have an insidious onset with subtle clinical manifestations. Consequently, lumbar puncture is warranted to obtain a diagnostic specimen of cerebrospinal fluid when there are complaints of headache or changes in mental status. Treatment with fluconazole[49] is an alternative to conventional therapy with amphotericin B plus flucytosine[50].

### PARASITIC INFECTION

The principle parasitic infection of liver transplant recipients has been *Pneumocystis carinii* pneumonia (PCP). PCP has not been found in some smaller series of transplant patients[2,6], but most larger series have reported an incidence of 1–11%[1,3,4,27]. The onset of PCP has been as early as 20 days after OLT[1], but cases generally have occurred several months after transplantation[1,4,27]. The frequency of PCP has been sufficiently low that prophylaxis is not routinely administered at many centers. *Toxoplasma gondii*, a protozoan pathogen of particular importance in cardiac transplant recipients, rarely has been reported in liver transplant patients[1,51]. Chagas disease, caused by *Trypanasoma cruzi* has been transmitted by donor kidneys and may eventually be transmitted to liver transplant recipients via the organ or blood transfusion.

### SUMMARY

Infection following OLT is a major problem that has been characterized only during the past few years. Serious infection occurs in about two-thirds of patients, and the risk of infection is greatest during the first month after transplantation[1,2,4,18]. During the first two or three weeks, most infections are bacterial and arise in the abdomen. The usual pathogens are aerobic enteric gram-negative bacilli and hard-to-treat bacteria such as enterococci, coagulase-negative staphylococci, and *Pseudomonas* that generally are outside the spectrum of the cephalosporin used for prophylaxis. To a lesser extent, *Candida* also is a significant abdominal pathogen during the first month or two. Beginning about three weeks after transplantation, CMV infection becomes increasingly important while the frequency of bacterial and fungal infection decreases. Beyond the second or third month after transplantation, the overall risk of infection declines, and the array of possible problems at that stage includes community-acquired bacterial pneumonia, cholangitis, CMV disease, and *Pneumocystis carinii* pneumonia.

Initial empiric treatment of suspected bacterial infection during the first few weeks after OLT should be aggressive because of the patient's immunosuppressed condition and the high frequency of primary or secondary bacteremia. The regimen selected should be based on three considerations: the "usual" pathogens following OLT, the likely pathogens following the type of anti-bacterial prophylaxis given to the patient, and the degree of risk from ongoing nosocomial problems such as legionellosis[18] or aspergillosis[45]. One regimen to consider for infections following conventional cefotaxime prophylaxis is the combination of a ureidopenicillin with tobramycin. Whatever empiric regimen is begun, it should be accompanied by vigorous diagnostic efforts to identify abscess or other problems that may require surgical intervention.

With regard to prophylaxis, promising options are available, though incompletely studied, for prevention of bacterial, fungal (yeast), and CMV infection. Selective bowel decontamination, directed toward elimination from gut of aerobic gram-negative bacilli and yeast, appears to decrease the risk of post-transplant infections due to those pathogens. Confirmation of efficacy in a randomized controlled trial is needed, since the only reported experience to-date is with a selected sub-group of patients who had a single transplant procedure, had not required antibiotics for at least two weeks prior to transplantation, and were stable enough to be on the SBD regimen pre-operatively for a mean of two weeks while active donor search proceeded[4,9]. Possible modifications of the SBD regimen include shorter pre- and post-transplant duration or use of alternative anaerobe-sparing agents such as quinolones. For CMV prophylaxis, the most economical approach may be oral acyclovir, which has shown promise in renal transplant recipients. Trials of oral acyclovir during the first two months after liver transplantation appear warranted, and use of ganciclovir for brief periods when there is early evidence of active viral replication should be considered.

### *References*

1. Kusne, S., Dummer, J.S., Singh, N., Iwatsuki, S., Makowka, L., Esquivel, C., Tzakis, A.G. and Starzl, T.E., Ho, M. (1988) Infections after liver transplantation: an analysis of 101 consecutive cases. *Medicine*, **67**, 132–143
2. Colonna, J.O. II, Winston, D.J., Brill, J.E., Goldstein, L.I., Haff, M.P., Hiatt, J.R., Quinones-Baldrich, W., Ramming, K.P. and Busuttil, R.W. (1988) Infectious complications in liver transplantation. *Arch. Surg.*, **123**, 360–364
3. Ascher, N.L., Stock, P.G., Bumgardner, G.L., Payne, W.D. and Najarian, J.S. (1988) Infection and rejection of primary hepatic transplant in 93 consecutive patients treated with triple immunosuppressive therapy. *Surg. Gynecol. Obstet.*, **167**, 474–484
4. Paya, C.V., Hermans, P.E., Washington, J.A. II, Smith, T.F., Anhalt, J.P., Wiesner, R.H. and Kromm, R.A.F. (1989) Incidence, distribution, and outcome of episodes of infection in 100 orthotopic liver transplantations. *Mayo Clin. Proc.*, **64**, 555–564
5. George, D.L., Arnow, P.M., Fox, A., Baker, A.L., Thistlethwaite, J.R., Emond, J.C., Whitington, P.F. and Broelsch, C.E. Bacterial infections complicating liver transplantation: epidemiology and risk factors. *Rev. Infect. Dis.* (in press)
6. Krom, R.A.F., Wiesner, R.H., Rettke, S.R., Ludwig, J., Southorn, P.A., Hermans, P.E. and Taswell, H.F. (1989) The first 100 liver transplantations at the Mayo Clinic. *Mayo Clin. Proc.* **64**, 84–94
7. Busuttil, R.W., Colonna, J.O. II, Hiatt, J.R., Brems, J.J., El Khoury, G., Goldstein, L.I., Quinones-Baldrich, W.J., Abdul-Rasool, I.H. and Ramming, K.P. (1987) The first 100 liver transplants at UCLA. *Ann Surg*, **206**, 387–399
8. Cuervas-Mons, V., Millan, I., Gavaler, J.S., Starzl, T.E. and Van Thiel, D.H. (1986) Prognostic value of pre-operatively obtained clinical and laboratory data in predicting survival following orthotopic liver transplantation. *Hepatology*, **6**, 922–927
9. Wiesner, R.H., Herman, P.E., Rakela, J., Washington, J.A. II, Perkins, J.D., DiCecco, S. and Krom, R. (1988) Selective bowel decontamination to decrease gram-negative aerobic bacterial and *Candida* colonization and prevent infection after orthotopic liver transplantation. *Transplantation*, **45**, 570–574
10. Van der Waaj, D. (1982) Colonization resistance of the digestive tract: clinical consequences and implications. *J. Antimicrob. Chemother*, **10**, 263–270
11. Singh, N., Dummer, J.S., Kusne, S., Breinig, M.K., Armstrong, J.A., Makowa, L., Starzl, T.E. and Ho, M. (1988) Infections with cytomegalovirus and other herpesviruses in 121 liver transplant recipients: transmission by donated organ and the effect of OKT3 antibodies. *J. Infect. Dis.*, **158**, 124–131
12. Breing, M.K., Zitelli, B., Starzl, T.E. and Ho, M. (1987) Epstein-Barr virus, cytomegalovirus, and other viral infections in children after liver transplantation. *J. Infect. Dis.*, **156**, 273–279
13. Gorensek, M.J., Carey, W.D., Vogt, D. and Goormastic, M. (1990) A multivariate analysis of risk factors for cytomegalovirus infection in liver-transplant recipients. *Gastroenterol*, **98**, 1326–1332
14. Stratta, R.J., Shaefer, M.S., Markin, R.S., Wood, P., Kennedy, E.M., Langas, A.N., Reed, A.C., Woods, G.L., Donovan, J.P., Pillen, T.J., Duckworth, R.M. and Shaw, B.W., Jr. (1989) Clinical patterns of cytomegalovirus disease after liver transplantation. *Arch. Surg.*, **124**, 1443–1449
15. Paya, C.V., Hermans, P.E., Wiesner, R.H., Ludwig, J., Smith, T.F., Rakela, J. and Krom,

R.A.F. (1989) Cytomegalovirus hepatitis in liver transplantation: prospective analysis of 93 consecutive orthotopic liver transplantations. *J. Infect. Dis.*, **160**, 752–758
16. Snover, D.C., Hütton, S., Balfour, H.H. Jr. and Bloomer, J.R. (1987) Cytomegalovirus infection of the liver in transplant recipients. *J. Clin. Gastroenterol*, **9**, 659–665
17. Alexander, J.A., Cuellar, R.E., Fadden, R.J., Genovese, J.J., Gavalier, J.S. and Van Thiel, D.H. (1988) Cytomegalovirus infection of the upper gastrointestinal tract before and after liver transplantation. *Transplantation*, **46**, 378–382
18. Jacobs, F., Van de Stadt, J., Bourgeois, N., Struelens, M.J., De Prez, C., Adler, M., Thys, J.P. and Gelin, M. (1989) Severe infections early after liver transplantation. *Transplant Proc.*, **21**, 2271–2273
19. O'Grady, J.G., Alexander, G.J.M., Sutherland, S., Donaldson, P.T., Harvey, F., Portmann, B., Calne, R.Y., and Williams, R. (1988) Cytomegalovirus infection and donor/recipient HLA antigens: interdependent co-factors in pathogenesis of vanishing bile duct syndrome after liver transplantation. *Lancet*, **2**, 302–305
20. Haagoma, E.B., Klompmaker, I.J., Grond, J., Bijleveld, C.M.E., The, T.H., Schirm, J., Gips, C.H. and Slooff, M.J.H. (1987) Herpes virus infections after orthotopic liver transplantation. *Transplant Proc.*, **19**, 4054–4056
21. Grundy, J.E., Lui, S.F., Super, M., Berry, N.J., Sweny, P., Fernando, O.N., Moorhead, J. and Griffiths, P.D. (1988) Symptomatic cytomegalovirus infection in seropositive kidney recipients: reinfection with donor virus rather than reactivation of recipient virus. *Lancet*, **11**, 132–135
22. Paya, C.V., Hermans, P.E., Smith, T.F., Rakela, J., Wiesner, R.H., Krom, R.A.F., Torres, V.E., Sterioff, S. and Wilkowske, C.J. (1988) Efficacy of ganciclovir in liver and kidney transplant recipients with severe cytomegalovirus infection. *Transplantation*, **46**, 229–234
23. Harbison, M.A., DeGirolami, P.C., Jenkins, R.L. and Hammer, S.M. (1988) Ganciclovir therapy of severe cytomegalovirus infections in solid-organ transplant recipients. *Transplantation*, **46**, 82–88
24. Bowden, R.A., Sayers, M., Fluornoy, N., Newton, B., Banaji, M., Thomas, E.D. and Meyers, J.D. (1986) Cytomegalovirus immune globulin and seronegative blood products to prevent primary cytomegalovirus infection after marrow transplantation. *N. Engl. J. Med.*, **314**, 1006–1010
25. Meyers, J.D., Reed, E.C., Shep, D.H., Thornquist, M., Dandliker, P.S., Vicary, C.A., Fluornoy, N., Kirk, L.E., Kersey, J.H., Thomas, E.D. and Balfour, H.H. Jr. (1988) Acyclovir for prevention of cytomegalovirus infection and disease after allogeneic marrow transplantation. *N. Engl. J. Med.*, **318**, 70–75
26. Balfour, H.H. Jr., Chace, B.A., Stapleton, J.T., Simmons, R.L. and Fryd, D.S. (1989) A randomized placebo-controlled trial of oral acyclovir for the prevention of cytomegalovirus disease in recipients of renal allografts. *N. Engl. J. Med.*, **320**, 1381–1387
27. Andrews, W., Fyock, B., Gray, S., Coln, D., Hendrickse, W., Siegel, J., Belknap, B., Hogge, A., Benser, M., Kennard, B., Stewart, S. and Albertson, N. (1987) Pediatric liver transplantation: the Dallas experience. *Transplant Proc.*, **19**, 3267–3276
28. Randhawa, P.S., Markin, R.S., Starzl, T.E. and Demetris, A.J. (1990) Epstein-Barr virus-associated syndromes in immunosuppressed liver transplant recipients. *Am. J. Surg. Pathol.*, **14**, 538–547
29. Ho, M., Jaffe, R., Miller, G., Breinig, M.K., Dummer, S., Makowka, L., Atchison, R.W., Karrer, F., Nalesnik, M.A. and Starzl, T.E. (1988) The frequency of Epstein-Barr virus infection and associated lymphoproliferative syndrome after transplantation and its manifestations in children. *Transplantation*, **45**, 719–727
30. Koneru, B., Jaffe, R., Esquivel, C.O., Kunz, R., Todo, S., Iwatsuki, S. and Starzl, T.E. (1987) Adenoviral infections in pediatric liver transplant recipients. *JAMA*, **258**, 489–492
31. Portmann, B., O'Grady, J., Williams, R. (1986) Disease recurrence following orthotopic liver transplantation. *Transplant Proc.*, **18** (suppl. 4), 136–141
32. Colledan, M., Grendele, M., Gridelli, B., Rossi, G., Fassati, L.R., Ferla, G., Doglia, M., Gislon, M. and Galmarini, D. (1989) Long-term results after liver transplantation in B and delta hepatitis. *Transplant Proc.*, **21**, 2421–2423
33. Demetris, A.J., Jaffe, R., Sheahan, D.G., Burnham, J., Spero, J., Iwatsuki, S., Van Thiel, D.H. and Starzl, T.E. (1986) Recurrent hepatitis B in liver allograft recipients. *Am. J. Pathol.*, **125**, 161–172
34. Starzl, T.E., Demetris, A.J. and Van Theil, D. (1989) Liver transplantation (second of two parts). *N. Engl. J. Med.*, **321**, 1092–1099

35. Lauchart, W., Muller, R. and Pichlmayr, R. (1987) Long term immunoprophylaxis of hepatitis B virus reinfection in recipients of human liver allografts. *Transplant Proc.*, **19**, 4051–4053
36. Rakela, J., Wooten, R.S., Batts, K.P., Perkin, J.D., Taswell, H.F. and Krom, R.A.F. (1989) Failure of interferon to prevent recurrent hepatitis B infection in hepatic allograft. *Mayo Clin. Proc.* **64**, 429–432
37. Agnes, S., Avolio, A.W., Magalini, S.C., Nanni, G., Foco, M., Serino, F., Hassan, G., Marinucci, G., Boldrini, G. and Castagneto, M. (1989) Results of liver transplantation for hepatitis delta disease without immunoprophylaxis. *Transplant Proc.*, **21**, 2426–2428
38. Fagan, E., Yousef, G., Brahm, J., Garelick, H., Mann, G., Wolstenholme, A., Portmann, B., Harrison, T., Mowbray, J.F., Mowat, A., Zuckerman, A. and Williams, R. (1990) Persistence of hepatitis A virus in fulminant hepatitis and after liver transplantation. *J. Med. Virol.*, **30**, 131–136
39. Wall, W.J., Duff, J.H., Ghent, C.N., Stiller, C.R., Keown, P.A. and Kutt, J.L. (1985) Liver transplantation: the initial experience of a Canadian center. *Can. J. Surg.*, **28**, 286–289
40. Grendele, M., Gridelli, B., Colledan, M., Rossi, G., Fassati, L.R., Ferla, G., Lunghi, G. and Galmarini, D. (1989) Hepatitis C virus infection and liver transplantation. *Lancet*, **2**, 1221–1222
41. Tzakis, A.G., Arditi, M., Whitington, P.F., Yanaga, K., Esquirel, C., Andrews, W.A., Makowka, L., Malatak, J., Freese, D.K., Stock, P.G., Ascher, N.L., Johnson, F.L., Broelsch, C.E. and Starzl, T.E. (1988) Aplastic anemia complicating orthotopic liver transplantation for non-A, non-B hepatitis. *N. Engl. J. Med.*, **319**, 393–396
42. Kusne, S., Dummer, J.S., Singh, N., Makowka, L., Esquivel, C., Starzl, T.E., Ho, M. (1988) Fungal infections after liver transplantation. *Transplant Proc.*, **20**, 650–651
43. Wajszczuk, C.P., Dummer, J.S., Ho, M., Van Thiel, D.H., Starzl, T.E., Iwatsuki, S. and Shaw, B. Jr. (1985) Fungal infections in liver transplant recipients. *Transplantation*, **40**, 347–353
44. Tollemar, J., Ericzon, B.-G., Holmberg, K. and Andersson, J. (1990) The incidence and diagnosis of invasive fungal infections in liver transplant recipients. *Transplant Proc.*, **22**, 242–244
45. Schroter, G.P.J., Hoelscher, M., Putnam, C.W., Porter, K.A. and Starzl, T.E. (1977) Fungus infections after liver transplantation. *Ann. Surg.*, **186**, 115–122
46. Boon, A.P., Adams, D.H., Buckels, J. and McMaster, P. (1990) Cerebral aspergillosis in liver transplantation. *J. Clin. Pathol.*, **43**, 114–118
47. Martinez, A.J. and Puglia, J. (1988) The neuropathology of liver, heart, and heart-lung transplantation. *Transplant Proc.*, **20**, (suppl. 1), 806–809
48. Viviani, M.A., Tortorano, A.M., Langer, M., Almaviva, M., Negri, C., Cristina, S., Scoccia, S., DeMaria, R., Fiocchi, R., Ferrazzi, P., Goglio, A., Gavazzeni, G., Faggian, G., Rinaldi, R. and Cadrobbi, P. (1989) Experience with itraconzole in cryptococcosis and aspergillosis. *J. Infection.*, **18**, 151–165
49. Robinson, P.A., Knirsch, A.K. and Joseph, J.A. (1990) Fluconazole for life-threatening fungal infections in patients who cannot be treated with conventional antifungal agents. *Rev. Infect. Dis.*, **12**, (suppl. 3) S349–S363
50. Bennett, J.E., Dismukes, W.E., Duma, R.J., Medoff, G., Sande, M.A. Gallis, H., Leonard, J., Fields, B.T., Bradshaw, M., Haywood, H., McGee, Z.A., Cate, T.R., Cobbs, C.G., Warner, J.F. and Alling, D.W. (1979) A comparison of amphotericin B alone and combined with flucytosine in the treatment of cryptococcal meningitis. *N. Engl. J. Med.*, **301**, 126–131
51. Salt, A., Sutehall, G., Sargaison, M., Woodward, C., Barnes, N.D., Calne, R.Y. and Wreghitt, T.G. (1990) Viral and *Toxoplasma gondii* infections in children after liver transplantation. *J. Clin. Pathol*, **43**, 63–67

*(Accepted by S. Bengmark on 10 September 1990)*

### INVITED COMMENTARY

Recent advances in organ preservation, refinements in surgical technique, and the routine use of veno-venous bypass has made orthotopic liver transplantation (OLT) a relatively safe and common procedure today. Dr. Arnow's review emphasizes the need to focus on infection as the major cause of morbidity and mortality in these patients. The timing and causes of bacterial, fungal and viral infections include reports from major centers encompassing a vast experience. The additional need for immunosuppression during this period not only complicates management but also sets the stage for subsequent viral illness.

Antibiotic prophylaxis has proven its importance in lowering the rate of postoperative infections in general surgical patients[1]. This approach in patients undergoing OLT is less clear, and requires special definition with regard to considerations of critically ill preoperative patients, prolonged operative time, and specific upper gastrointestinal flora. Selective bowel decontamination, as reported by the Mayo Clinic group seems to decrease serious gram negative infection in the first 3 postoperative weeks, however its acceptance will not be universal until randomized prospective studies are done. The need for such a prolonged and extensive regimen may not be necessary for all patients. ICU bound pretransplant patients may well benefit from combined oral and intravenous prophylaxis at the time of admission, while stable out patient candidates could simply be prepared from the time of donor identification.

CMV continues to cause significant morbidity in OLT patients and often follows bursts of increased immunosuppression required to manage rejection. It has even been implicated in the development of chronic rejection. The successful use of prophylactic acyclovir in renal recipients[2] has naturally extended to those undergoing OLT and prospective studies are underway. Gancyclovir is now available for treatment of established infections and should be considered for future prophylactic trials in high risk patients. Not one case of PCP pneumonia has been reported in over 600 patients undergoing transplantation at the University of Pittsburgh since routine PCP prophylaxis was begun in December 1988. This is compared to 11 episodes with 3 deaths in 101 patients undergoing OLT prior to this time[3]. Considering the risk/benefit ratio, PCP prophylaxis should be routinely administered to all patients.

The management of established infections is equally difficult with choice of appropriate antibiotics based on culture profiles and with minimal nephrotoxicity. Meticulous attention to surgical technique and hemostasis is of utmost importance initially. Routine microbial surveillance postoperatively and careful attention to technical complications which frequently lead to infections, combined with timely reductions in immunosuppression cannot be over emphasized.

### *References*

1. Burnakis, T.G. (1984) Surgical Antimicrobial Prophylaxis: Principles and Guidelines. *Pharmacother*, **4**(5), 248–271
2. Balfour, H.H. Jr., Chace, B.A., Stapleton, J.T. and Simmons, R.L. A Randomized Placebo-Controlled Trial of Oral Acyclovir for the Prevention of Cytomegalovirus disease in Recipients of renal allografts. *N. Engl. J. Med.*, **320**, 1381–1387
3. Kusne, S., Dummer, S., Singh, N. *et al.* (1988) Infections after Liver Transplantation. An analysis of 101 Consecutive Cases. *Medicine*, **67**(2), 132–143

F. Maureen Martin
Richard Simmons
Department of Surgery
University of Pittsburgh

